# The outcomes in nephrotoxicity among therapies utilizing three distinct polymyxins: a systematic review and network meta-analysis

**DOI:** 10.3389/fmed.2026.1869822

**Published:** 2026-06-22

**Authors:** Huan Zhang, Hong Chen, Zheng Zhang, Haiqing Shi, Jianbo Li, Xue Zhang, Sipan Wang, Xuelian Liao

**Affiliations:** 1 Department of Critical Care Medicine, West China Hospital of Sichuan University, Chengdu, Sichuan, China; 2 Department of Critical Care Medicine, West China Tianfu Hospital of Sichuan University, Chengdu, Sichuan, China

**Keywords:** colistimethate sodium, colistin sulfate, nephrotoxicity, network meta-analysis, polymyxin B

## Abstract

**Objectives:**

Polymyxin B (PMB), colistin sulfate, and colistimethate sodium (CMS) are key therapies for multidrug-resistant Gram-negative infections in critically ill patients. However, their comparative nephrotoxicity remains unclear with inconsistent evidence; therefore, this study used a network meta-analysis to systematically compare their renal safety and related clinical outcomes.

**Methods:**

Studies comparing PMB, colistin sulfate, and CMS were identified through systematic searches of PubMed, EMBASE, the Cochrane Library, Web of Science, CNKI, and Wanfang databases up to April 4, 2026. A frequentist network meta-analysis was conducted to estimate the relative risk of nephrotoxicity among the three polymyxin formulations by integrating direct and indirect evidence within a unified analytical framework. Secondary outcomes included 28-day mortality, microbiological eradication, and length of hospital stay. Data were synthesized using fixed- or random-effects models as appropriate, based on the degree of between-study heterogeneity.

**Results:**

Compared with patients receiving colistin sulfate, those receiving CMS showed higher estimated odds of nephrotoxicity in both the network analysis (OR 3.65, 95% CI 1.31–10.19) and the direct pairwise comparison (OR 2.47, 95% CI 1.44–4.22). In contrast, patients receiving PMB did not show a statistically significant difference in nephrotoxicity compared with those receiving colistin sulfate (OR 0.99, 95% CI 0.68–1.43). In direct comparisons, colistin sulfate cohort was also associated with a lower risk of nephrotoxicity than PMB cohort (OR 0.46, 95% CI 0.28–0.77). Age- and serum albumin-adjusted analysis and subgroup analysis yielded broadly similar results. For secondary outcomes, patients receiving colistin sulfate or PMB showed higher estimated odds of 28-day mortality compared with those receiving CMS, whereas no statistically significant difference in 28-day mortality was observed between colistin sulfate and PMB cohort. Across treatment comparisons, no significant differences were identified in bacterial clearance or length of hospital stay.

**Conclusion:**

This network meta-analysis of observational cohort studies suggests that colistin sulfate may be associated with a lower risk of nephrotoxicity than CMS- and PMB-based therapy. Nevertheless, given the heterogeneity across studies and the potential for residual confounding inherent in observational evidence, these findings should be interpreted cautiously and regarded as hypothesis-generating until further evaluated in well-designed prospective cohort studies or randomized controlled trials.

**Systematic review registration:**

https://www.crd.york.ac.uk/PROSPERO/, identifier CRD420261322516.

## 1 Introduction

The rapid emergence and global spread of multidrug-resistant Gram-negative bacteria (MDR-GNB) represent a major public health concern, with a growing burden of limited therapeutic options and increased mortality. As the development of novel antibiotics remains insufficient, polymyxins—previously abandoned due to concerns regarding nephrotoxicity and neurotoxicity—have re-emerged as important treatment options for treating severe MDR-GNB infections (1).

Polymyxins are a class of cyclic cationic polypeptide antibiotics that exert bactericidal activity by binding to lipopolysaccharides and phospholipids, thereby disrupting the outer membrane of gram-negative bacteria. In clinical practice, three formulations are used: polymyxin B sulfate (PMB), colistimethate sodium (CMS), and colistin sulfate (2). PMB and colistin sulfate are administered as active compounds, whereas CMS is an inactive prodrug that requires *in vivo* hydrolysis to generate the active colistin form. Although these agents share similar core structures and antimicrobial spectra, they differ in pharmacokinetic and metabolic properties, which may influence toxicity profiles. PMB undergoes limited renal clearance and is primarily eliminated via non-renal pathways, whereas CMS is predominantly renally excreted and partially converted to colistin *in vivo*. Colistin sulfate, approved for clinical use in China since 2018, shares structural similarities with PMB but may have a distinct safety profile related to formulation and metabolism (3).

Existing studies yield inconsistent findings regarding the relative nephrotoxicity and clinical outcomes of different polymyxin formulations. Direct comparative evidence for colistin sulfate versus PMB or CMS remains limited, and head-to-head meta-analytic data across all three formulations are scarce. Therefore, this systematic review and meta-analysis aimed to comprehensively evaluate and compare the incidence of nephrotoxicity and related clinical outcomes among patients receiving PMB-, CMS-, and colistin sulfate-based therapies.

## 2 Materials and methods

This network meta-analysis was conducted in accordance with the PRISMA extension statement for network meta-analyses and recommendations from the Cochrane Collaboration (4). The protocol was prospectively registered in PROSPERO under registration number CRD420261322516. Because this study was based solely on previously published data and did not involve any direct human or animal experimentation, ethical approval and informed consent were not required.

### 2.1 Literature search strategy

A systematic literature searched PubMed, EMBASE, the Cochrane Library, the Web of Science (WOS), China National Knowledge Infrastructure (CNKI), and Wanfang Standards Database (WFPD) from the database inception to April 04, 2026. The language was restricted to English and Chinese. The search strategy combined terms related to the population, polymyxin-based interventions, and study design (cohort studies, randomized controlled trials), The search was performed using subject headings, keywords, and MeSH term searches for “colistin,” “polymyxin B,” “colistimethate sodium,” “acute kidney injury,” and “nephrotoxicity.” We utilized the medical subject heading (MeSH) term retrieval, free-text terms, and Boolean logic operation were used to search for original articles in the PubMed database. Literature searches in EMBASE, the WOS, and the Cochrane Library were conducted by using keywords for retrieval.

### 2.2 Inclusion and exclusion strategy

Studies were considered eligible if they compared the incidence of nephrotoxicity among different polymyxin-based treatment cohorts. Studies were eligible if they met the following criteria: (1) Population: hospitalized adult patients (aged ≥18 years) with infection, such as pneumonia, bacteremia, abdominal infections, urinary tract infections, or other infectious diseases. (2) Interventions: any of the three main interventions; (3) The study outcomes must include nephrotoxicity as an endpoint; (5) Study design: both prospective and retrospective cohort studies, as well as randomized controlled trials, were included in the analysis. Studies were excluded if they met any of the following criteria: methodological quality score < 4 on the Newcastle-Ottawa Scale (NOS); case reports, reviews, systematic reviews, comments, conference abstracts, or editorials; duplicate publications; studies with incomplete or missing original data; and animal or *in vitro* studies.

First, Two independent investigators (ZH and CH) screened all the articles based on the title, then assessed the abstracts according to a standardized form. Finally, target data were carefully collected through the full-text review. Discrepancies were resolved through discussion or, when necessary, consultation with a third author (LX L), who provided guidance and made the final determination.

### 2.3 Outcomes and definitions

The primary outcome of this study was the incidence of nephrotoxicity associated with polymyxin-based therapy. Secondary outcomes included all-cause mortality, clinical cure rate, microbiological response, and length of hospital stay.

Nephrotoxicity was defined according to the criteria used in each included study, most commonly based on the Kidney Disease: Improving Global Outcomes (KDIGO) (5) or Risk, Injury, Failure, Loss, and End—stage kidney disease (RIFLE) classification systems (6). In studies where formal criteria were not specified, nephrotoxicity was identified based on changes in serum creatinine (SCr), typically defined as an increase of ≥0.5 mg/dL or ≥50% from baseline during polymyxin treatment. When multiple definitions were reported, the most stringent or KDIGO-aligned criterion was adopted to ensure consistency across studies.

Mortality was extracted as 28- or 30-days mortality, according to the time point reported in the included studies. Microbiological eradication was defined as the elimination of the causative pathogen on follow-up microbial culture after the use of antibiotics.

### 2.4 Data extraction

The following data were extracted from each included study: (1) basic study information, including the first author’s name, publication year, country, and study design; (2) Infection status: infection site, type of bacteria, and severity of the illness; (3) Intervention details, including polymyxin formulation (colistin sulfate, CMS, or PMB) and treatment regimen; and (4) Clinical outcomes, including the incidence of nephrotoxicity, 28-day mortality, microbiological eradication rate, and length of hospital stay.

### 2.5 Methodological quality assessment

The quality of all the included observational cohort studies was assessed by the Newcastle-Ottawa Scale (NOS) (7). The checklist of NOS contains three parameters: the rule of population collection, comparability of each cohort group, and the exposure or results of interest in each cohort study. Studies with NOS scores ≥4 were considered to be of acceptable methodological quality and were included in the meta-analysis. Randomized controlled trials, if present, were assessed according to the Cochrane risk-of-bias tool.

### 2.6 Statistical method

Statistical analyses were performed within a frequentist framework. Study arm reporting outcomes for PMB, CMS, or colistin sulfate were entered separately. Pairwise meta-analyses were first performed using a random-effects model, followed by a multiple-treatment network meta-analysis that integrated direct and indirect evidence across the three interventions. Covariate-adjusted network meta-analysis were performed to evaluate the effect of age, serum albumin and APACHE II score on renal function. Subgroup analyses were performed according to the infection type. Heterogeneity in pairwise comparisons was assessed using the chi-square test and the inconsistency statistic (*I*^2^). Heterogeneity was considered significant when *p* < 0.1 and *I*^2^ > 50%. A random-effects model was used in the presence of moderate or high heterogeneity (*I*^2^ > 25% and *p* < 0.1); otherwise, a fixed-effects model was used. Dichotomous outcomes were summarized using odds ratios (ORs) with 95% confidence intervals (CIs), and statistical significance was set at *p* < 0.05.

Sensitivity analyses were conducted by excluding studies with small sample sizes to assess the robustness of the findings. Consistency between direct and indirect evidence was evaluated using both global inconsistency tests and node-splitting analyses, with *p* < 0.05 indicating potential inconsistency. All analyses were performed using R software (version 4.3.3, e.g., netmeta package).

## 3 Results

### 3.1 Literature retrieval

**FIGURE 1:**
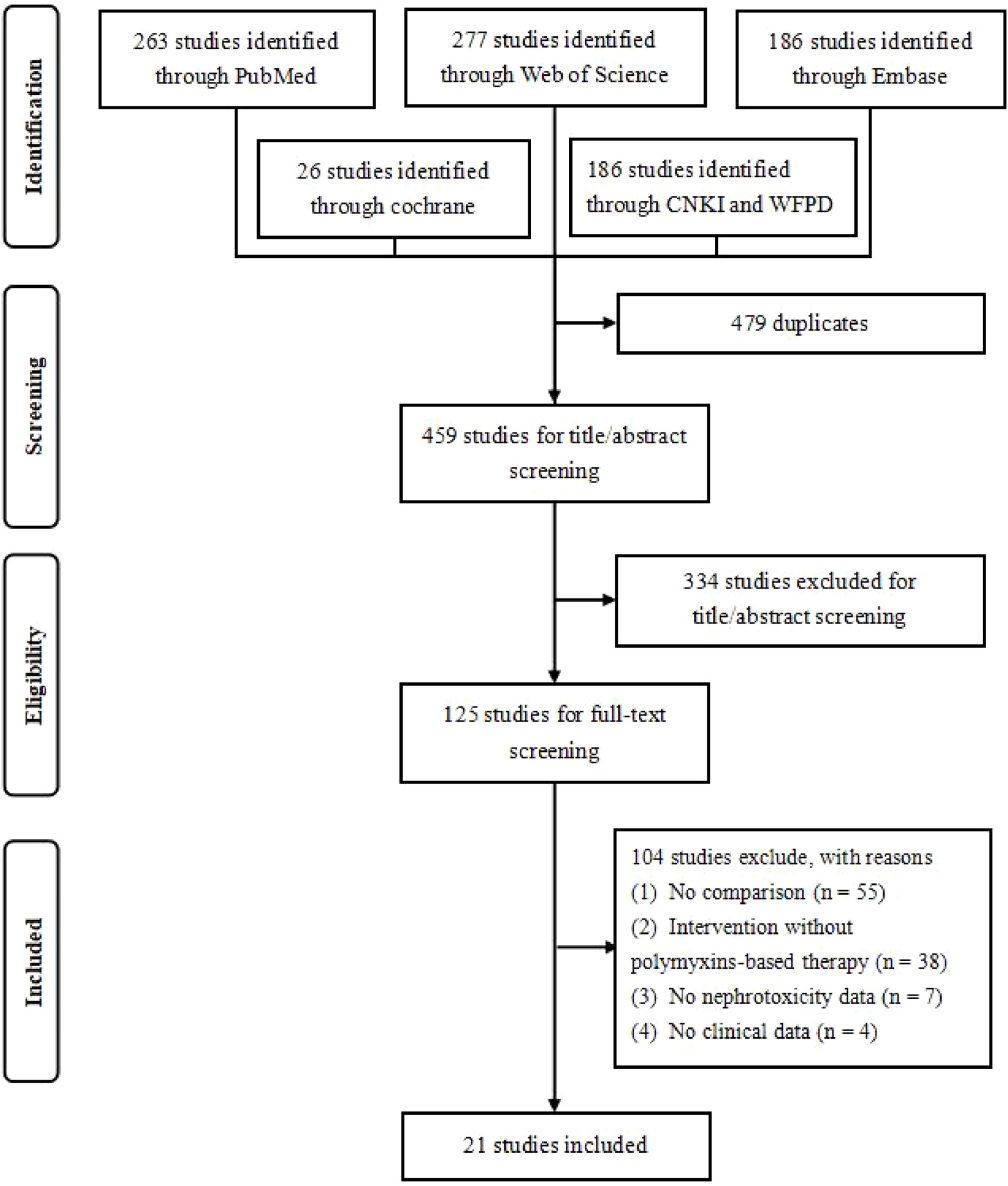
The PRISMA flow diagram of this review.

A total of 938 studies were initially identified: 263 from *PubMed*, 186 from *EMBASE*, 277 from *Web of Science (WOS)*, 26 from *the Cochrane Library*, and 186 from *CNKI* and *Wanfang Data (WFPD)*. After removing 479 duplicates, 459 records remained for screening. Based on titles and abstracts, 395 studies were excluded for irrelevance. The full texts of the remaining 125 articles were then carefully reviewed. Of these, 55 lacked a comparative design, 38 did not involve polymyxin-based interventions, and 11 had incomplete or missing clinical data. Finally, 21 studies, including 19 retrospective and 2 prospective cohort studies, met all inclusion criteria and were included in the meta-analysis (8–28). The study selection process is presented in the PRISMA flow diagram in Figure 1.

### 3.2 Quality and characteristics of included studies

All 21 included studies were rated as moderate-quality studies according to the NOS criteria, with scores ranging from 4.5 to 6.5. The characteristics and methodological quality assessment of each study are shown in Supplementary Table 1. The included studies in this meta-analysis were published between 2009 and 2025, and all were observational cohort studies. Geographically, five studies were conducted in Brazil, eleven in China, and two in the United States, with one study each from the United Kingdom, Italy, and Buffalo, New York.

The clinical use of the three polymyxin formulations varied across studies and included monotherapy with different administration routes as well as combination therapy with other antimicrobial agents. All studies reported the incidence of nephrotoxicity. Seven studies applied the KDIGO criteria, eight used the RIFLE criteria, and six defined nephrotoxicity based on changes in baseline serum creatinine (SCr). Detailed baseline characteristics of the included studies are summarized in Table 1.

**TABLE 1:** The basic characteristics of studies included in this meta-analysis.

Author	Year of publication	Region	study period	Cohort study type	Feature of patients	AKI in PMB cohort	AKI in colistin cohort	AKI in CMS cohort	Other outcomes*	Albumin reported	Main infection site
Qian Zeng	2025	China	June 2020 to November 2024	Retrospective	CR-GNB	29/189	9/89	15/47	1,2,4	Yes	Pneumonia
Fang Huang	2025	China	Dec 2021 and Dec 2023	Retrospective	CR-GNB	36/173		26/87	1,2	Yes	Blood
Yuanfang Qin	2025	China	July 2018 and July 2023	Retrospective	MDR-GNB	183/354	38/208	–	–	No	Mixed
Ping Yang	2025	China	Jan 2023 to Dec 2023	Retrospective	NA		8/76	8/76	1	No	Mixed
Bu Wei	2025	China	Jan 2020–Jun 2024	Retrospective	CR-GNB	27/95	18/95	–	1, 3, 4	No	Pneumonia
Yijing Zhang	2025	China	Aug 2021–Dec 2023	Retrospective	ICU-CR-GNB	17/59	18/59		1, 2	Yes	Mixed
Zhang, Y.	2024	China	Oct 2021–Nov 2022	Retrospective	CR-GNB	52/101	19/101		1	No	Mixed
Yang, Q.-j.	2024	China	Jan 2017–Jan 2024	Retrospective	ICU	24/114	8/114		1, 2	Yes	Mixed
Wu, T	2024	US	Jan 2004–Dec 2022	Retrospective	NA	120/131		60/578	NA	No	Mixed
Vieceli, T.	2024	Brazil	Apr 2016–Sep 2021	Retrospective	CRKP-BSI	31/56		22/44	1	No	Blood
Balli, F. N.	2024	Turkiye	Jan 2020–Jul 2022	Retrospective	NA	33/95		50/95	1	No	Mixed
Di Liu.	2024	China	Jan 2020–Sep 2022	Retrospective	CR-GNB	0/68	2/52		1, 4, 5	No	Pneumonia
Wang, J.	2023	China	Nov 2021–Nov 2022	Retrospective	ICU_CR-GNB	10/68	5/36		1, 2, 4	No	Mixed
Simon, V.	2023	Brazil	2018–Oct 2019	prospective	MDR-GNB	25/54		59/108	1, 4, 5	No	Mixed
Garcia, R. C. L.	2023	Brazil	2014–2021	Retrospective	CRE	117/212		22/47	1	No	Blood
Truong, C. B.	2021	US	2004–2020	Retrospective	NA	229/1740		21/252	1	No	Mixed
Quintanilha, J. C. F.	2019	Brazil	Aug 2010–Jul 2013	Retrospective	ICU	28/51		22/51	1	No	Mixed
Ritesh, A.	2018	India	Jul 2016–Jun 2017	prospective	ICU	32/51		10/61	NA	No	Mixed
Crass, R. L.	2017	UK	Not available	Clinical Therapeutics	Without CF	21/49		73/145	1, 2	Yes	Mixed
					With CF	10/29		57/191	1, 2	Yes	Mixed
S, A. D.	2013	Brazil	1996–2004	retrospective	ICU	28/67		64/106	1,3	No	Mixed
Oliveira, M. S.	2009	Buffalo	2008–2010	Retrospective	CRAB	8/41		10/41	2	No	Mixed

### 3.3 Primary outcomes: the incidence of nephrotoxicity

**FIGURE 2:**
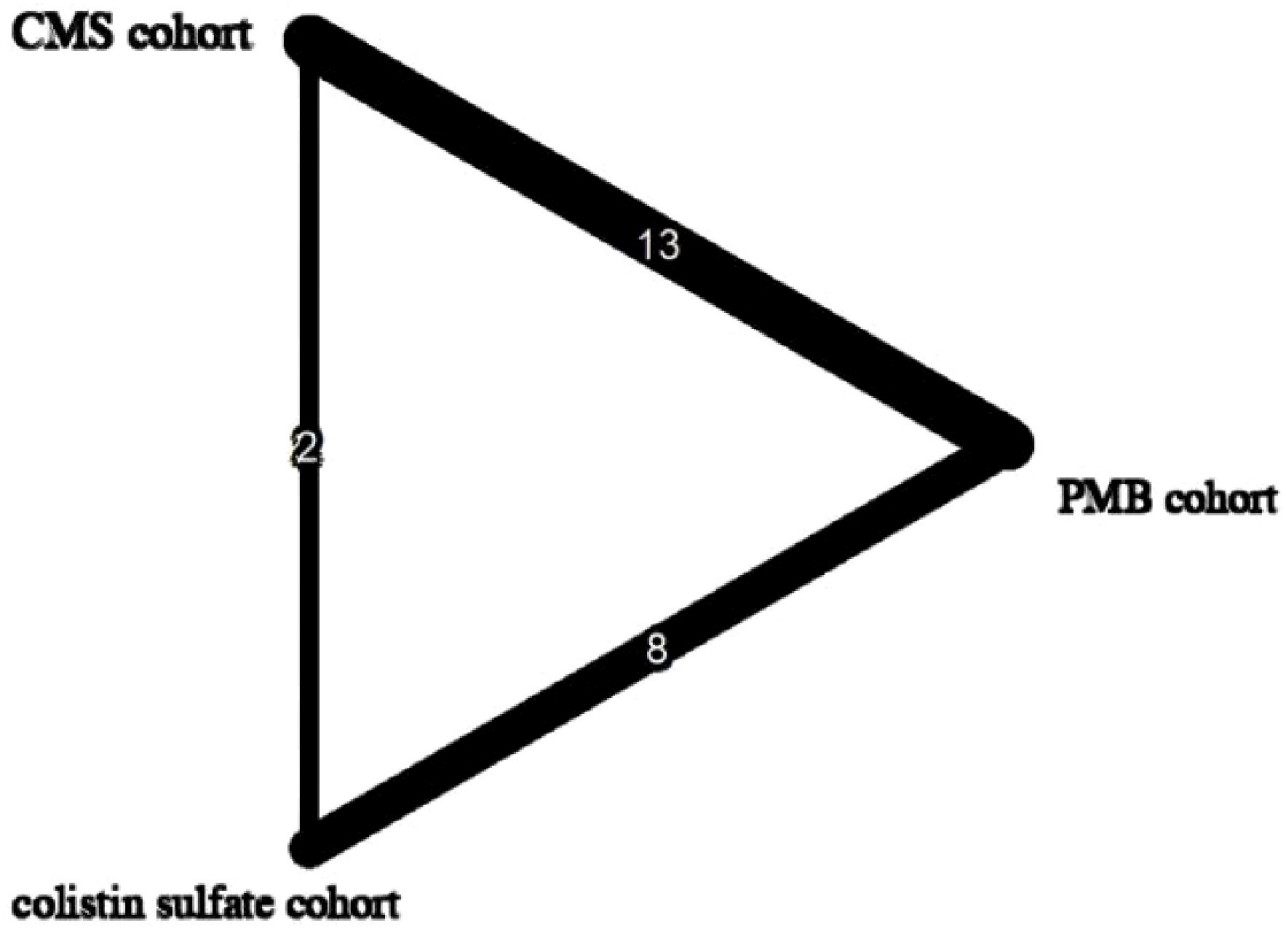
Network analysis of nephrotoxicity across colistin sulfate, CMS, and PMB cohorts. The network diagram illustrated the direct comparisons among the three antibiotic cohorts: colistin sulfate, CMS, and PMB. Nodes represented the treatment cohorts, and the connecting lines indicated available head-to-head comparisons. The numbers on the lines denoted the number of studies included for each direct comparison, with line thickness proportional to the number of studies.

Based on the predefined search strategy, 21 studies comparing three polymyxin regimens were included, contributing 23 two-arm comparisons, as shown in Figure 2. Among the included patients, 3,787 received PMB-based therapy, 829 patients received colistin sulfate-based therapy, and 1,933 received CMS-based therapy. Because substantial heterogeneity was observed (*I*^2^ = 73%, τ^2^ = 0.337), the network meta-analysis was conducted using a random effects model.

Compared with colistin sulfate-based therapy, CMS-based regimens was associated with a significantly higher risk of nephrotoxicity in both the network meta-analysis and direct comparison analyses (network meta-analysis OR 3.65, 95% CI 1.31–10.19; direct comparison OR 2.47, 95% CI 1.44–4.22). PMB-based therapy showed no statistically significant difference in nephrotoxicity risk compared with CMS-based therapy (network meta-analysis OR 0.99, 95% CI 0.68–1.43; direct comparison OR 1.04, 95% CI 0.73–1.48). In addition, colistin sulfate-based regimens was associated with a lower risk of nephrotoxicity compared with PMB-based therapy (network meta-analysis OR 0.46, 95% CI 0.28–0.77; direct comparison OR 0.42, 95% CI 0.27–0.67). These findings were summarized in the league table (Table 2), with network meta-analysis estimates shown in the lower triangle and direct comparison estimates shown in the upper triangle.

**TABLE 2:** Network meta-analysis league table showing ORs (95% CIs) for nephrotoxicity with PMB, CMS, and colistin (random-effects model).

Relative cohort	CMS cohort	Colistin sulfate cohort	PMB cohort
CMS cohort		2.47(1.44–4.22)	1.04(0.73–1.48)
Colistin sulfate cohort	3.65(1.31–10.19)		0.42(0.27–0.67)
PMB cohort	0.99(0.68–1.43)	0.46(0.28–0.77)	

Lower triangle: network meta-analysis (column vs. row); Upper triangle: direct comparisons (row vs. column).

Substantial heterogeneity was observed across studies, which may have reflected differences in study populations, treatment regimens, and definitions of nephrotoxicity. However, no statistically significant inconsistency between direct and indirect evidence was detected (*Q* = 0.50, *P* = 0.48), suggesting that the network estimates were broadly coherent.

### 3.4 Sensitivity analysis

To assess the robustness of the primary findings, sensitivity analyses were conducted by sequentially excluding studies with a sample sizes of fewer than 100 patients. Using a random-effects network meta-analysis, the results remained consistent with the primary analysis for nephrotoxicity risk across polymyxin regimens.

In the direct comparisons, the CMS cohort was associated with a significantly higher risk of nephrotoxicity than the colistin sulfate cohort (OR = 3.65, 95% CI 1.28–10.36) and the PMB cohort (OR = 2.35, 95% CI 1.32–4.17). No statistically significant difference was observed between colistin sulfate and PMB cohort (OR = 0.99, 95% CI 0.68–1.45). The network meta-analysis showed similar directional effects. Colistin sulfate- and PMB- based theraapies were associated with a lower risk of AKI than CMS-based therapy (OR = 0.42, 95% CI 0.26–0.69 for colistin; OR = 0.47, 95% CI 0.28–0.78 for PMB). No statistically significant difference was found between colistin sulfate- and PMB-based therapy (OR = 0.97, 95% CI 0.66–1.43).

### 3.5 Covariate-adjusted network meta-analysis

The relative treatment estimates remained largely unchanged after adjustment for age, as shown in Supplementary Table 2. The adjusted estimates were as follows: CMS-based therapy versus colistin sulfate-based therapy, OR 2.47 (95% CI 1.44–4.22); CMS-based therapy versus PMB-based therapy, OR 3.65 (95% CI 1.30–10.18); and PMB-based therapy versus colistin sulfate-based therapy, OR 0.42 (95% CI 0.27–0.67). These findings suggest that age may not have been a major study-level effect modifier of nephrotoxicity risk across the included studies. They also suggest that the observed differences in nephrotoxicity among polymyxin agents were not influenced by variations in mean age across studies. However, because the included studies were observational and the adjustment was based on study-level covariates, these findings should be interpreted cautiously.

Because serum albumin may influence drug toxicity, a serum albumin-adjusted network meta-analysis was performed using data from seven studies that reported baseline albumin values, as shown in Supplementary Figure 1. In the adjusted network estimates, the CMS- based cohort was associated with a significantly higher risk of renal dysfunction than the colistin sulfate cohort, with a relative effect of 2.83 (95% CI: 1.37–5.83). In contrast, the colistin sulfate cohort showed a significantly lower risk compared with PMB based cohort, with a relative effect of 0.52 (95% CI: 0.29–0.95). The comparison between CMS based cohort and PMB based cohort did not reach statistical significance, with a relative effect of 1.48 (95% CI: 0.86–2.55). Direct comparison results were generally consistent for the comparison between CMS based cohort and colistin based cohort, showing a significantly higher risk in CMS based cohort than in colistin based cohort, with a relative effect of 4.17 (95% CI: 1.21–14.33). However, direct comparisons between CMS-based and PMB based cohort, and between colistin sulfate cohort and PMB based cohort, were not statistically significant. which was shown in Supplementary Table 3.

Regarding critical illness status, nine cohorts reported the APACHE II scores and contributed two-arm comparisons, as shown in Supplementary Figure 2. After adjustment for APACHE II score, no statistically significant differences in renal function impairment were observed among CMS-based, colistin sulfate-based, and PMB-based cohorts in either the network meta-analysis or direct comparisons, as shown in Supplementary Table 4. Only five studies reported SOFA score, therefore, SOFA score was not included as a covariate in the network meta-analysis.

### 3.6 Subgroup network meta-analysis

Regarding pathogen type, 13 cohorts reported data for patients with MDR-GBN infections, as shown in Supplementary Figure 3. Therefore, a subgroup network meta-analysis was performed in this population. In the random-effects network meta-analysis, significant differences in AKI risk were observed among CMS-, colistin-, and PMB-based cohorts. The network estimates showed that CMS- based therapy was associated with a significantly higher risk of AKI compared with colistin sulfate-based therapy, with an effect estimate of 2.42 (95% CI: 1.23–4.76). In contrast, colistin sulfate-based therapy was associated with a significantly lower AKI risk compared with PMB-based therapy, with an effect estimate of 0.54 (95% CI: 0.32–0.91). No statistically significant difference was observed between CMS- and PMB- based cohort, with an effect estimate of 1.30 (95% CI: 0.79–2.12). The results from direct comparisons were generally consistent with the network estimates, which was shown in Supplementary Table 5.

### 3.7 Treatment ranking

*P*-scores ranged from 0 to 1, with higher values indicating a greater probability of being the best treatment in terms of lower nephrotoxicity risk in this study. Colistin sulfate cohort had the highest ranking for safety with respect to AKI (*P*-score = 1.00), followed by PMB cohort (*P*-score = 0.29), whereas CMS cohort ranked lowest (*P*-score = 0.21), indicating the greatest risk of nephrotoxicity. The ranking results were consistent with random-effects models.

Overall, these findings indicate that colistin sulfate may be associated with a lower risk of AKI than CMS- and PMB-based therapy, whereas no statistically significant difference was observed between CMS- and PMB-based cohorts. The concordance between network meta-analysis and direct comparisons supports that these estimates were broadly consistent.

### 3.8 Secondary outcomes

#### 3.8.1 28 -day mortality

Based on the screened outcomes, we analyzed 28-day mortality, including studies reporting 30-day mortality, microbiological eradication, and length of hospital stay, all of which were considered clinically relevant outcomes.

A network meta-analysis was performed to compare 28-day mortality among patients receiving CMS-, colistin sulfate-, and PMB-based therapy. The analysis included 11 PMB-based arms, 6 colistin sulfate-based arms, and 9 CMS-based arms, as shown in Supplementary Figure 4. Random-effects estimates were reported because heterogeneity was observed across studies (*I*^2^ = 40.3%, τ^2^ = 0.35).

In the network estimates, the colistin sulfate cohort differed significantly from the CMS cohort in 28-day mortality (OR 0.34, 95% CI 0.15–0.80). The PMB cohort also differed from the CMS cohort (OR 0.65, 95% CI 0.42–0.99). Direct comparisons were broadly consistent with the network estimates. The direct comparison between colistin sulfate- and CMS-based regimen showed an OR of 0.56 (95% CI 0.33–0.94), while PMB cohort versus CMS cohort showed an OR of 1.15 (95% CI 0.76–1.74). When comparing colistin sulfate cohort with PMB cohort, no statistically significant difference in 28-day mortality was observed (network estimate OR 0.76, 95% CI 0.47–1.22; direct estimate OR 1.00, 95% CI 0.64–1.58).

Overall, the analysis suggested differences in 28-day mortality among the three treatment cohorts, particularly for colistin sulfate- versus CMS-based therapy. No statistically significant difference were observed between colistin sulfate and PMB, suggesting broadly comparable mortality estimates between these two cohorts. These results was shown in Table 3.

**TABLE 3:** League table showing network and direct comparison odds ratios (OR, 95% CI) for 28-day mortality across CMS-, colistin-, and PMB-based cohorts (Random-effects model).

Comparison	CMS based cohort	Colistin sulfate cohort	PMB cohort
CMS based cohort		0.56 [0.33–0.94]	1.15 (0.76–1.74)
Colistin sulfate cohort	0.34 [0.15–0.78]		1.00 (0.64–1.58)
PMB based cohort	0.65 [0.42–0.99]	0.76 [0.47–1.22]	

Lower triangle shows network meta-analysis estimates (column vs. row); upper triangle shows direct comparisons (row vs. column). OR < 1 favors the column treatment for lower mortality.

#### 3.8.2 Bacteria eradication

In the pairwise comparisons among the three treatment regimens, the analysis included 8 cohorts, as shown in Supplementary Figure 5, no statistically significant difference in bacterial clearance were observed. The comparison between the CMS-based and the colistin sulfate-based cohort (OR = 0.48, 95% CI 0.10–2.31). Similarly, no significant difference was observed between the CMS-based and the PMB- based cohort (OR = 0.52, 95% CI 0.14–1.98), or between the colistin sulfate-based and the PMB-based cohorts (OR = 1.09, 95% CI 0.33–3.55).

Consistent findings were observed in the corresponding comparisons. The colistin sulfate-based cohort did not differ significantly from the CMS-based cohort (OR = 0.23, 95% CI 0.02–3.07), and PMB-based cohort did not differ significantly from the CMS-based cohort (OR = 0.68, 95% CI 0.15–3.01). Likewise, no statistically significant difference was observed between the PMB-based and colistin sulfate-based cohort (OR = 0.91, 95% CI 0.25–3.30).

Overall, no clear superiority was observed among the three treatment strategies in terms of bacterial clearance. All 95% confidence intervals crossed 1, indicating that the network meta-analysis did not support a statistically significant advantage of any regimen, as shown in Table 4.

**TABLE 4:** League table showing network and direct comparison odds ratios (OR, 95% CI) for bacterial clearance across CMS-, colistin sulfate-, and PMB-based cohorts (Random-effects model)

Comparison	CMS based cohort	Colistin sulfate based cohort	PMB based cohort
CMS based cohort		0.48 [0.01–2.31]	0.52 (0.14–1.98)
Colistin sulfate based cohort	0.23 [0.02–3.07]		1.09 (0.33–3.55)
PMB based cohort	0.68 (0.15–3.01)	0.91 (0.25–3.30)	

Lower triangle shows network meta-analysis estimates (column vs. row); upper triangle shows direct comparisons (row vs. column).

#### 3.8.3 Duration of hospitalization stay

A total of 10 cohorts contributed to the pairwise comparisons among the three treatment regimens, as shown in Supplementary Figure 6. The network meta-analysis using a random-effects model (tau = 6.47; *I*^2^ = 97.30%) evaluated hospital length of stay across CMS-, colistin-, and PMB-based cohorts. As shown in Table 5, neither colistin sulfate- nor PMB-based cohorts showed statistically significant differences in length of stay compared with CMS, with network mean differences of −3.54 days (95% CI −11.01 to 3.94) for colistin sulfate cohort vs. CMS cohort and −1.45 days (95% CI −7.84 to 4.94) for PMB cohort vs. CMS cohort. Comparisons between colistin sulfate cohort and PMB cohort also did not indicate significant differences (network MD 2.09 days, 95% CI −3.28 to 7.46), as shown in Table 5.

**TABLE 5:** League table of mean differences (MD, 95% CI) for hospital length of stay (days) across CMS-, colistin sulfate-, and PMB-based cohorts (random-effects network meta-analysis).

Comparison	CMS cohort	Colistin sulfate cohort	PMB cohort
CMS cohort		−3.54 (−11.01; 3.94)	−1.45 (−7.84; 4.94)
Colistin sulfate cohort	−7.24 (−20.05; 5.57)		2.09 (−3.28; 7.46)
PMB cohort	−0.29 (−7.46; 6.88)	1.34 (−4.43; 7.11)	

Lower triangle: network meta-analysis estimates (column vs. row); Upper triangle: direct comparisons (row vs. column).

## 4 Discussion

In this network meta-analysis of 21 cohort studies, we compared the nephrotoxicity associated with three polymyxin antibiotics: PMB, CMS, and colistin sulfate. The findings suggest that colistin sulfate-based regimens may be associated with a lower risk of nephrotoxicity than CMS- and PMB- based therapies, with variation observed in both direct and indirect comparisons. In addition, colistin sulfate- and PMB-based therapies appeared to be associated with higher 28-day mortality than CMS-based therapy, with the increase reaching statistically significant for colistin sulfate cohort. For microbiological eradication and length of hospital stay no clear superiority was observed among the three treatment groups. Given the heterogeneity across the included observational cohort studies, larger well-designed cohort studies and, ideally, randomized controlled trials are needed to confirm and further refine these findings.

Polymyxins remain important treatment options for multidrug-resistant Gram-negative bacterial infections, but nephrotoxicity continues to limit their clinical use. Reported incidence of the three polymyxin-associated nephrotoxicity vary across studies and among different formulations. These differences may be partly explained by pharmacokinetic and elimination characteristics. PMB and colistin sulfate are extensively reabsorbed by the renal tubules, whereas CMS is predominantly eliminated through renal excretion and requires conversion to its active colistin in the kidneys. Consequently, CMS may exposes the kidneys to both the prodrug and active colistin, which circulates in the plasma and may accumulate in renal tissue (29). Unlike CMS, both PMB and colistin sulfate exert antibacterial activity without requiring conversion, and their metabolites are not primarily eliminated via the kidneys (30).

Selecting the optimal polymyxin regimen for CR-GNB pneumonia requires careful consideration of both efficacy and toxicity. PMB has been supported by stronger guideline recommendations (31). Since publication of the guidelines, additional studies have compared the nephrotoxicity of PMB, CMS, and colistin sulfate.

Comparative evidence regarding CMS, colistin sulfate, and PMB remains inconsistent. Several studies reported a significantly higher incidence of nephrotoxicity with CMS than with PMB (32, 33), whereas others observed no significant difference in AKI risk (23). Previous studies have also reported favorable clinical efficacy and safety for colistin sulfate (28). In addition, two independent studies reported comparable AKI between PMB and colistin sulfate cohorts (14).

This meta-analysis suggests that colistin sulfate may have a more favorable renal safety profile than PMB or CMS. Unlike previous studies, this review included only cohort studies with direct comparisons among PMB-, CMS- and colistin sulfate-based therapies. However, the included studies differed in their definitions of AKI, dosing strategies, concomitant medications, and study periods, which may have contributed to between-study heterogeneity and should be considered when interpreting the findings.

After adjustment for age, protein level and APACHE II score, the findings suggest that adjustment for clinical covariates may influence the estimated renal safety profile of polymyxin-related treatments. Adjustment for age and protein level suggested a higher risk of renal dysfunction with CMS-based therapy than with colistin sulfate-based therapy, whereas adjustment for APACHE II score attenuated this association and rendered all comparisons non-significant. Future studies with larger sample sizes, standardized definitions of renal dysfunction, and more complete adjustment for baseline renal function, disease severity, dosage, treatment duration, and concomitant nephrotoxic agents are needed to clarify the comparative renal safety of CMS-, colistin sulfate-, and PMB-based therapies.

In addition, an apparently paradoxical pattern is observed for colistin sulfate, which appears to show a lower nephrotoxicity signal but a higher estimated 28-day mortality compared with CMS. This discrepancy should be interpreted cautiously because nephrotoxicity and mortality are likely influenced by different clinical mechanisms, and lower kidney toxicity does not necessarily translate into improved survival. Mortality in this population may be driven primarily by baseline disease severity, infection site, pathogen virulence, organ dysfunction, and supportive care. In primary cohort studies (25, 28), patients receiving colistin sulfate appeared to have more severe infections, highlighting the need for cautious antibiotic selection and further trials to define optimal therapy for multidrug-resistant Gram-negative infections. What is more, evidence for colistin sulfate was mainly derived from Chinese studies, which may limit the generalizability of the mortality findings to other healthcare settings. A further possible explanation is competing risk: patients with early death may have had insufficient time for nephrotoxicity to develop or be documented, potentially leading to an apparently lower nephrotoxicity rate despite higher mortality. Therefore, this finding should be regarded as hypothesis-generating and interpreted in the context of observational study design and incomplete adjustment for baseline severity.

The meta-analysis has several limitations. First, substantial heterogeneity was present, which may be partly attributable to the observational design of all included studies. In addition, dosing strategies and administration protocols for the three polymyxins varied across studies. The definition of nephrotoxicity was also not uniform. Although these definitions differed, each appeared to capture clinically relevant renal injury.

Administration route may have contributed to the observed heterogeneity. However, although several studies reported the proportion of patients receiving intravenous-only therapy or intravenous plus inhaled therapy, route-specific AKI outcomes were generally unavailable. and the influence of administration route on the pooled effect remains uncertain.

Furthermore, another important limitation is the restricted geographic applicability of the evidence on colistin sulfate. Because colistin sulfate is mainly used in China, the included evidence for this agent was derived predominantly from Chinese populations and healthcare settings. Therefore, the comparative nephrotoxicity and mortality estimates for colistin sulfate may not be directly generalizable to countries where colistin sulfate is unavailable or rarely used. Differences in patient characteristics, antimicrobial resistance patterns, dosing practice, renal monitoring, and supportive care may further limit external validity. Nevertheless, by comprehensively analyzing eligible cohort studies published in English and Chinese, our review provides a more complete assessment of polymyxin-associated nephrotoxicity and clinical efficacy, and may offer useful insight insights for clinical practice.

## 5 Conclusion

This network meta-analysis of observational cohort studies suggests that colistin sulfate may be associated with a lower risk of nephrotoxicity than CMS- and PMB-based therapy. Nevertheless, given the heterogeneity across studies and the potential for residual confounding inherent in observational evidence, these findings should be interpreted cautiously and regarded as hypothesis-generating until further evaluated in well-designed prospective cohort studies or randomized controlled trials.

## Data Availability

The original contributions presented in this study are included in this article/Supplementary material, further inquiries can be directed to the corresponding author.
