# Supplementary material for: The outcomes in nephrotoxicity among therapies utilizing three distinct polymyxins: a systematic review and network meta-analysis
Source: Front Med (Lausanne). 2026 Jun 22;13:1869822. doi: 10.3389/fmed.2026.1869822 (PMC13333463; doi:10.3389/fmed.2026.1869822)
Supplement: Supplementary file 1 [file Supplementary_file_1.docx]

Supplementary Figure 1. Serum albumin-adjusted network meta-analysis of nephrotoxicity across colistin sulfate, CMS, and PMB cohorts.


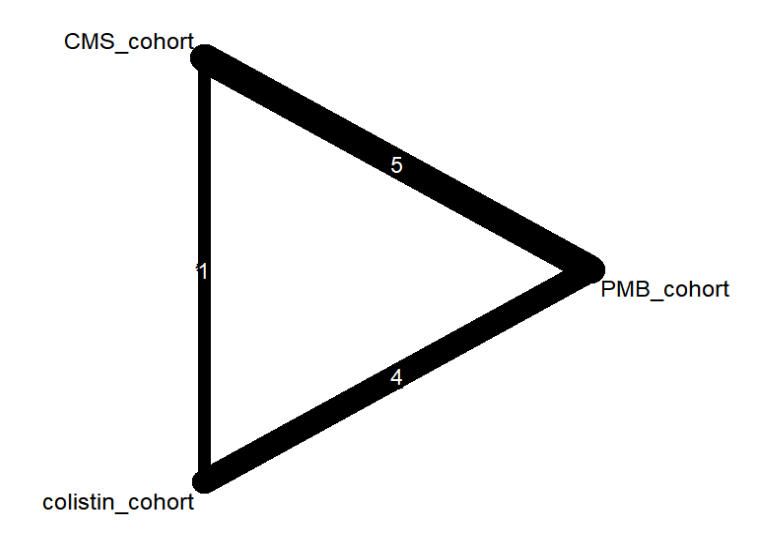


**Figure Legend:**
The network diagram illustrates direct comparisons among the three antibiotic cohorts: colistin sulfate, CMS, and PMB. Nodes represent the treatment cohorts, and the connecting lines indicate available head-to-head comparisons. The numbers on the lines denote the number of studies included for each direct comparison. Line thickness is proportional to the number of studies. PMB = polymyxin B; CMS = colistimethate sodium.

Supplementary Figure 2. APACHE II score-adjusted network meta-analysis of nephrotoxicity across colistin sulfate, CMS, and PMB cohorts.


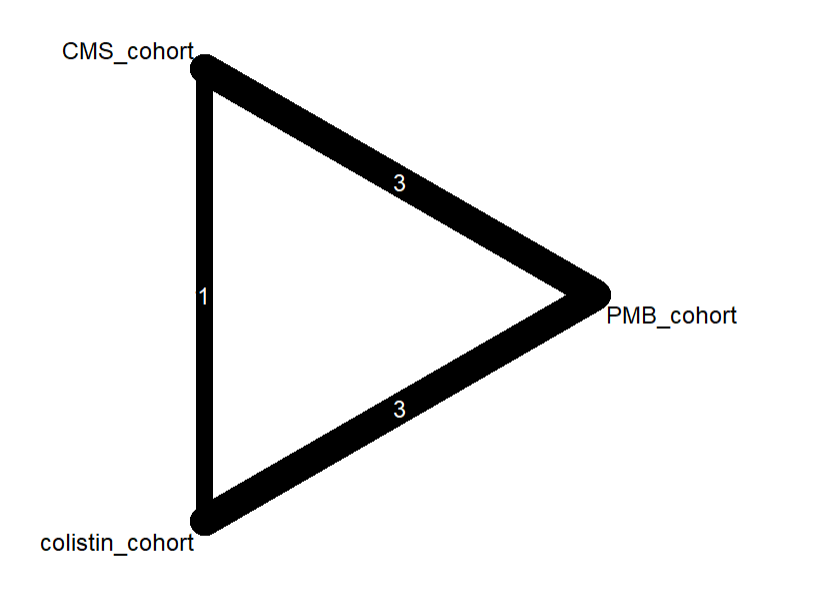


**Figure Legend:**
The network diagram illustrates direct comparisons among the three antibiotic cohorts: colistin sulfate, CMS, and PMB. Nodes represent the treatment cohorts, and the connecting lines indicate available head-to-head comparisons. The numbers on the lines denote the number of studies included for each direct comparison. Line thickness is proportional to the number of studies. PMB = polymyxin B; CMS = colistimethate sodium.

Supplementary Figure 3. **Network and direct comparison odds ratios (ORs) for AKI with PMB, CMS, and colistin** sulfate cohor**t in subgroup analyses according to MDR-GBN patients.**


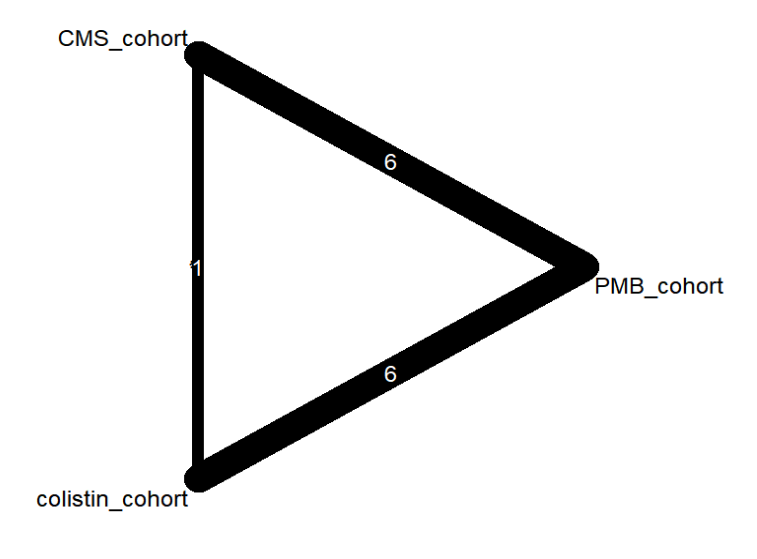


**Figure Legend:**
The network diagram illustrates direct comparisons among the three antibiotic cohorts: colistin sulfate, CMS, and PMB. Nodes represent the treatment cohorts, and the connecting lines indicate available head-to-head comparisons. The numbers on the lines denote the number of studies included for each direct comparison. Line thickness is proportional to the number of studies. PMB = polymyxin B; CMS = colistimethate sodium.

Supplementary Figure 4. Network analysis of 28-day mortality across colistin sulfate, CMS, and PMB cohorts.


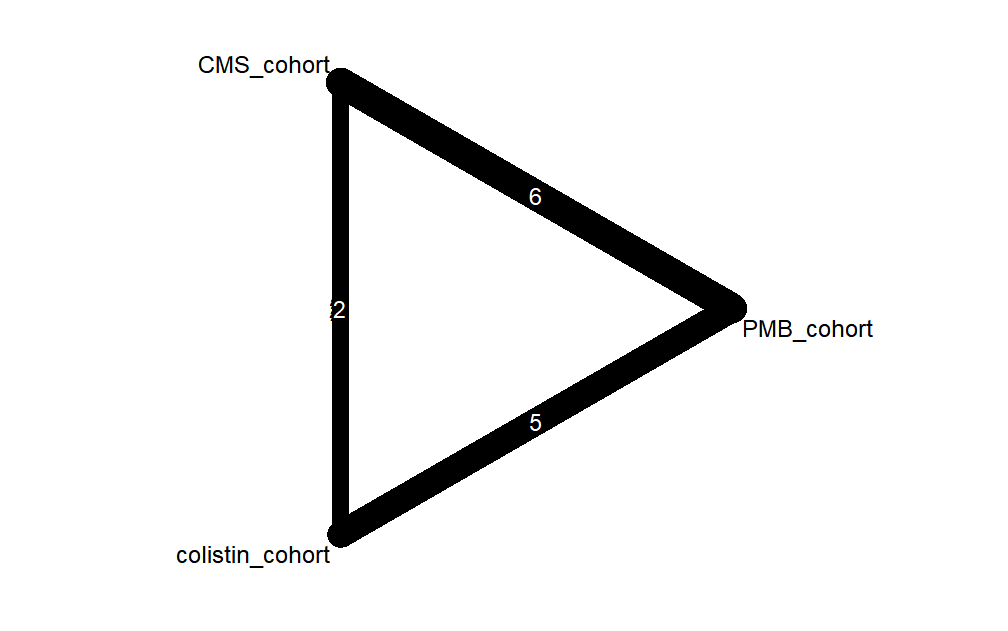


**Figure Legend:**
The network diagram illustrates direct comparisons among the three antibiotic cohorts: colistin sulfate, CMS, and PMB. Nodes represent the treatment cohorts, and the connecting lines indicate available head-to-head comparisons. The numbers on the lines denote the number of studies included for each direct comparison. Line thickness is proportional to the number of studies. PMB = polymyxin B; CMS = colistimethate sodium;

Supplementary Figure 5. Network analysis of bacteria eradication across colistin sulfate, CMS, and PMB cohorts.


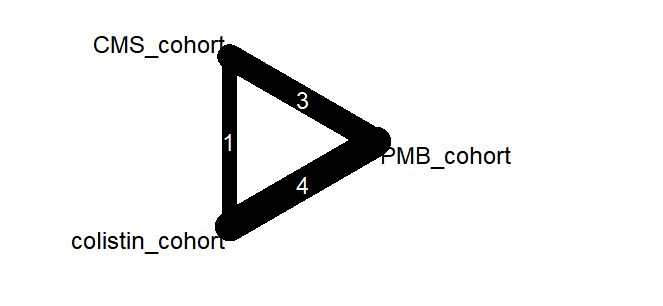


**Figure Legend:**
The network diagram illustrates direct comparisons among the three antibiotic cohorts: colistin sulfate, CMS, and PMB. Nodes represent the treatment cohorts, and the connecting lines indicate available head-to-head comparisons. The numbers on the lines denote the number of studies included for each direct comparison. Line thickness is proportional to the number of studies. PMB = polymyxin B; CMS = colistimethate sodium.

(3) Supplementary Figure 6. Network analysis of duration of hospitalization stay across colistin sulfate, CMS, and PMB cohorts.


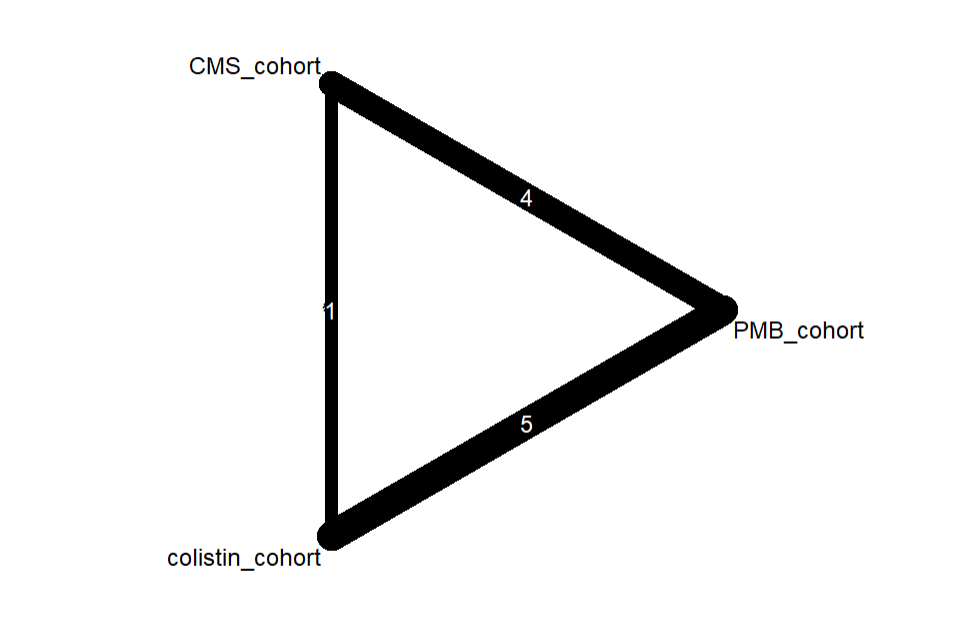


**Figure Legend:**
The network diagram illustrates direct comparisons among the three antibiotic cohorts: colistin sulfate, CMS, and PMB. Nodes represent the treatment cohorts, and the connecting lines indicate available head-to-head comparisons. The numbers on the lines denote the number of studies included for each direct comparison. Line thickness is proportional to the number of studies. PMB = polymyxin B; CMS = colistimethate sodium.

Supplementary Table 1 Quality of the included cohort studies (The Newcastle–Ottawa scale).

| **study** | **Representativeness of the exposed cohort** | **Selection of the nonexposed cohort** | **Ascertainment of exposure** | **Outcome not present at start** | **Comparability of cohorts** | **Assessment of outcome** | **follow-up longenough** | **Adequacy of follow-up** | **Total score** |
| --- | --- | --- | --- | --- | --- | --- | --- | --- | --- |
| Qian Zeng | 1 | 1 |  | 0.5 | 1 | 1 | 1 | 1 | 6.5 |
| Fang Huang | 1 | 1 |  | 0.5 | 1 | 1 | 1 | 1 | 6.5 |
| Yuanfang Qin | 1 | 1 |  | 0.5 | 1 | 1 | 1 | 1 | 5.5 |
| Ping Yang | 1 | 1 |  | 0.5 | 1 | 2 | 1 | 1 | 6.5 |
| Wei Bu | 1 | 1 |  | 0.5 | 1 | 2 | 1 | 1 | 6.5 |
| Bu W, Wang | 1 | 1 |  | 0.5 | 1 | 2 | 1 |  | 6.5 |
| Yijing Zhang | 1 | 1 |  | 0.5 | 1 | 2 | 1 |  | 6.5 |
| Zhang, Y. | 1 | 1 |  | 0.5 | 1 | 2 | 1 |  | 6.5 |
| Yang, Q.-j. | 1 | 1 |  | 0.5 | 1 | 1 |  |  | 4.5 |
| Wu, T | 1 | 1 |  | 0.5 | 1 | 2 |  |  | 5.5 |
| Vieceli, T. | 1 |  |  | 0.5 | 2 | 2 |  |  | 5.5 |
| Balli, F. N. | 1 |  |  | 1 | 2 | 1 |  |  | 5 |
| Di Liu. | 1 | 1 |  | 1 | 2 | 1 | 1 |  | 6 |
| Wang, J. | 1 |  |  | 1 | 2 | 1 |  |  | 5 |
| Simon, V. | 1 | 1 |  | 1 | 2 | 1 |  |  | 6 |
| Garcia, R. C. L. | 1 | 1 |  | 1 | 2 | 1 |  |  | 6 |
| Truong, C. B. | 1 | 1 |  | 0.5 | 2 | 1 |  |  | 5.5 |
| Quintanilha, J. C. F. | 1 | 1 |  | 0.5 | 2 | 1 |  |  | 5.5 |
| Ritesh, A. | 1 | 1 |  | 0.5 | 2 | 1 |  |  | 5.5 |
| Crass, R. L. | 1 | 1 |  | 0.5 | 2 | 1 |  |  | 5.5 |
| S, A. D. | 1 | 1 |  | 0.5 | 2 | 2 |  |  | 6.6 |
| Oliveira, M. S. | 1 | 1 |  | 0.5 | 2 | 1 |  |  | 5.5 |

Supplementary **Table 2. Network and direct comparison odds ratios (ORs) for AKI with PMB, CMS, and colistin** sulfate cohort**, adjusted for age (Random-effects model)**

| **Comparison** | **CMS cohort** | **Colistin sulfate cohort** | **PMB cohort** |
| --- | --- | --- | --- |
| **CMS cohort** |  | 0.99 [0.68–1.43] | 1.04 [0.73–1.48] |
| **Colistin sulfate cohort** | 2.47 [1.44–4.22] |  | 0.46 [0.28–0.77] |
| **PMB cohort** | 3.65 [1.30–10.18] | 0.42 [0.27–0.67] |  |

Supplementary **Table 3. Network and direct comparison odds ratios (ORs) for AKI with PMB, CMS, and colistin** sulfate cohort**, adjusted f**or serum albumin (Random-**effects model).**

| **Comparison** | **CMS cohort** | **Colistin sulfate cohort** | **PMB cohort** |
| --- | --- | --- | --- |
| **CMS cohort** |  | 4.17 [1.21–14.33] | 1.35 [0.75–2.46] |
| **Colistin sulfate cohort** | 2.83 [1.37–5.83] |  | 0.58[0.30–1.14] |
| **PMB cohort** | 1.48 [0.86–2.55] | 0.52 [0.29–0.95] |  |

Supplementary **Table 4. Network and direct comparison odds Ratios (ORs) for AKI with PMB, CMS, and colistin** sulfate cohort**, adjusted for APACHE II (Random-effects model).**

| **Comparison** | **CMS cohort** | **Colistin sulfate cohort** | **PMB cohort** |
| --- | --- | --- | --- |
| **CMS cohort** |  | 3.20 [0.53–19.29] | 0.70[0.32–1.53] |
| **Colistin sulfate cohort** | 1.58 [0.57–4.35] |  | 0.62[0.24–1.60] |
| **PMB cohort** | 0.80 [0.39–1.67] | 0.51 [0.22–1.20] |  |

Supplementary **Table 5. Network and direct comparison odds ratios (ORs) for AKI with PMB, CMS, and colistin** sulfate cohort **in subgroup analyses according to MDR-GBN patients (Random-effects model).**

| **Comparison** | **CMS cohort** | **Colistin sulfate cohort** | **PMB cohort** |
| --- | --- | --- | --- |
| **CMS cohort** |  | 4.17 [1.04–16.77] | 1.20[0.71–2.03] |
| **Colistin sulfate cohort** | 2.42 [1.23–4.76] |  | 0.59[0.33–1.03] |
| **PMB cohort** | 1.30 [0.79–2.12] | 0.53 [0.32–0.91] |  |
